# Debating secularism: A liberal cosmopolitan perspective

**DOI:** 10.3389/fsoc.2023.1113208

**Published:** 2023-03-02

**Authors:** Haldun Gülalp

**Affiliations:** Turkish Economic and Social Studies Foundation (TESEV), Istanbul, Türkiye

**Keywords:** secularism, liberalism, multiculturalism, state sovereignty, Tariq Modood, Talal Asad, Thomas More

## Abstract

In the classical notion of secularism, privatization of religion is an essential component of freedom and equality between citizens, so that rights are granted to individuals rather than to communities. The currently dominant objections to this notion in the literature are the multiculturalist thesis, primarily expounded by Tariq Modood, and the critique of secularism through the “genealogical” method, associated with Talal Asad and his followers. This article critically assesses these objections and defends the classical notion of secularism from a liberal cosmopolitan perspective. The argument that the classical notion perfectly addresses the questions of freedom of conscience and diversity of belief is further supported by reference to an ignored source, Thomas More's *Utopia*.

## Introduction

In the 1980s and 1990s, Islamists in Turkey used to cite postmodernist literature to critique the secular modern state and argue that historically Muslim societies had implemented multicultural models for freedom of religion, as in the *contract* between the Prophet and non-Muslim religious communities back in Medina and in the *millet* system of the Ottoman Empire (Gülalp, 1997). Sultan Mahmud II (1808–1839) was a pioneering modernizer who introduced reforms that culminated in the *Tanzimat* (Lewis, 2001), which was decried by Islamists for declaring (albeit only nominally and inconsequentially) equality between Ottoman subjects of different faiths, implying the end of Muslim superiority. Among the Sultan's novelties was the abolition of distinct dress codes for members of different religious communities because, as he allegedly said, he wished to see religious differences between his subjects only when they were in their respective houses of worship (Quataert, 1997). For this aspiration, he came to be known among conservatives as the “*giaour*” (infidel) sultan.

The Sultan's aspiration contained a vision of “secularism,” where *privatization* of religion was the essential component of freedom and equality between citizens, and where rights were to be granted to individuals rather than to communities.^1^ There are currently two types of objections to this notion of secularism. Although cross references between these two literatures are non-existent, or minimal, they unite in the rejection of liberal individualism, on which the modern state is presumably based.

One is the multiculturalist thesis. Expounded most notably by Modood (2013, 2019), its key point of contention is that individuals are not isolated entities, for they are embedded in communities that give them their social and political identities and their sense of dignity. Accordingly, rights and recognition ought to be granted not to individuals but to communities. Multiculturalism, it is argued, would allow for the public and political representation of communal religious identities, whereas secularism does not adequately recognize the freedom of religion.

Seemingly opposed to this primordialist objection, though converging with it, is the postmodernist objection, which locates the problem in modernity itself. According to this critique, developed primarily by Asad (1993, 2003), secularism is a mode of sovereignty of the modern nation-state. Asad follows Foucault's genealogical method and replicates the latter's almost mirror-reflective reversal of the modernization paradigm of the 1950s by finding modernity at the source of all ills of society. He argues that “religion” has been defined, shaped, delineated and restricted by the modern state, belying its claims to liberal secularity or neutrality toward religion.

Both multiculturalists and postmodernists perceive secularism as suppression of freedom of religion. Yet, for both, the desired religious freedom implies *not* the freedom of the individual citizen to believe in a faith, and exercise it alone or in community with others, as in the original conception of modern secularism, but freedom *for* religion(s) as corporate entities, an arrangement that would ultimately empower the communal hierarchy and disempower the individual believer along with the non-believer.

## Multicultural secularism

The point that individuals are not free-floating entities but “culturally embedded” in their communities is made by many (e.g., Parekh, 2000), but a direct link to the debate on “secularism” is found primarily in the work of Tariq Modood, whose normative theory is based on two pillars. First is his concept of “moderate secularism,” which, strictly speaking, is independent of the question of religious diversity in society. The latter is addressed by the second pillar of his normative theory, the notion of “multicultural secularism.”

### Moderate secularism

Modood (2019, p. 195, *passim*) offers the following basic definition of “political secularism,” which accords with the one noted above: “politics or the state has a raison d'être of its own and should not be subordinated to religious authority, religious purposes or religious reasons.” He declares commitment to this concept, but also weakens it by introducing what he calls “state-religion connections” (SRCs) as a desirable feature of governing religion (Modood, 2019, Ch. 10). The existence of SRCs gives us “moderate secularism,” as opposed to the radical or exclusionary forms of secularism that deny any public or political appearance to religion in Modood's assessment, for which he cites France and Turkey as examples. SRCs may exist whether there is one or more religions in society. In case of religious diversity, “multiculturalism” is achieved by extending SRCs to other (recognized) religions.

It is questionable whether France and Turkey are proper examples of “radical secularism” (cf. Lemmens, 2019; Gülalp, 2022, respectively). Leaving that aside, the concept of “moderate secularism” itself must be questioned. Modood (2019, p. 180*ff* ) defines religion as a “public good,” that is, as beneficial to public life, and argues that the state ought to support religion and religious organizations, as well as allow them to contribute to society by taking over some of the tasks of the state and offer services in education, welfare, health care, and so on. He believes that this situation is not only prevalent in most of Europe but also normatively desirable.

Below, some examples of SRCs will show that they could hardly be described as beneficial to public life. Even at a conceptual level, though, if they empower religious communities and/or organizations they would disempower individual citizens. Religion may be considered a “public good” for the believers, but there are also unbelievers and agnostics in society, along with followers of a variety of religions that are not officially recognized. When religious bodies take over some social services that the state ought to provide to all citizens equally, they encroach upon the field of the secular state, weakening the foundation for freedom and equality that must exist between citizens irrespective of their religion or level of religiosity. Therefore, SRCs potentially violate Modood's concept of “political secularism” quoted above.

Modood duly notes that “if religious organizations are supported with public funds or tasked by the state to carry out some educational or welfare duties then they must be subject to certain requirements, such as equal access or non-discrimination” (Modood, 2019, p. 181). But it is not clear how this might work, considering Modood's expansion of his basic concept of “political secularism” to arrive at the following definition: “political authority does not rest on religious authority and the latter does not dominate political authority; each has considerable, though not absolute, autonomy” (Modood, 2019, p. 180). If the state interferes in the business of these religious bodies in order to ensure that they do not discriminate in favor of their own members or followers, and serve all citizens equally, it would be violating this principle of mutual autonomy.

In terms of the logic of secularism, however, the matter is much less complicated: the religious body may certainly organize collective worship, teach doctrine, and so on, in a way that does not disturb social peace; but beyond these activities, limited to matters of belief and conscience, it cannot have autonomy in matters concerning this-worldly affairs. That realm is the business of the (secular) democratic state, which is in principle answerable to *all* citizens, whereas the religious organization is not bound in any such fashion. The “mutual autonomy” can never be equally mutual as the secular state has the responsibility, and hence must have the authority, to ensure that the rules of democratic and civil conduct are not violated by organizations within its jurisdiction, including the religious ones.

Finally, moral values that derive from religious faith may be brought by believing citizens into political debate and negotiation (and thus count as “public good”), but cannot be turned into an imposition on others. In democratic deliberation, citizens may be inspired or motivated by their religious beliefs and certainly have the right to voice them publicly. But they must appeal to others through a language that will be clear to all and never express their own beliefs as something sacred to be respected by all. What equal citizens are obligated to mutually respect is the right to freedom of belief for each and all, but not the contents or tenets of the belief system itself. Modood's SRCs and moderate secularism, however, involve religion in political and state affairs in a significant divergence from the standard conception of normative secularism.

### Racializing religion

In Modood's own account (Modood, 2013, 2019), the second pillar of his normative theory is inspired by the 1990's literature on identity politics, particularly those by Kymlicka (1995) on “multiculturalism” and Young (1990) on “differentiated citizenship.” Yet, he notes that the category of religion is lacking in them and proposes to expand the framework. Defining religious identity as an ascribed attribute, equivalent to race or gender, he argues for building a multicultural system that includes the diverse religious communities in society (Modood, 2019, Chs. 6, 11, *passim*). There are two issues here, one to do with theory and the other with policy.

Beginning with theory, while Kymlicka is primarily concerned with ethno-linguistic diversity within national boundaries, Young is concerned with structural inequalities and cites the following social groups in a catalog of the oppressed and the excluded: “women, workers, Jews, blacks, Asians, Indians, Mexicans” (Young, 1995, p. 181), to which list she might have added the LGBTQ, physically challenged, and others. The only thing in common between these distinct social groups is that they all experience some form of discrimination, but they do so in different ways and for completely different reasons that have nothing to do with culture. Any cultural differences that may emerge originate from structural foundations, such as capitalism, colonialism, patriarchy, ableism, and so on, whether in accommodation or in protest to them. Under such circumstances, would there be any intrinsic value to preserving those cultures? Or, should we rather strive to end the structures of inequality that cause any differential cultural behaviors? Would the concepts of respecting identities and building multiculturalism even have any relevance in those circumstances? Given this context, the desire to assert and protect difference as a matter of “identity politics” would at best address the symptoms, but miss the sources of structural problems.

Religion, which may indeed be a cultural phenomenon worth preserving (by its believers), is therefore not congruous with these structural categories. Modood claims that religious identity is equivalent to race, gender, or sexual orientation insofar as one is born into a religious community, which is not a matter of choice, just as one may be born into an environment in which religious identity is a source of discrimination (Modood, 2019, p. 123–124, *passim*). But even if it were true that religious identity belongs to the category of ascriptive structural attributes, we still need to distinguish between opposition to discrimination and the demand to protect cultural authenticity. While the former is a condition for freedom and equality, the latter may not even be a realistic goal, as cultures are only imaginarily unified and constantly subject to change. Worse, the protected culture itself may be intrinsically oppressive, in which case the goal of opposing oppression and discrimination would itself be defeated. There is another big difference between religion and the structural categories listed above. It would be sexist, for example, to assert that women are naturally incapable of solving mathematical problems, and racist to claim that Blacks, Jews, or Turks are by essence illiberal. But it would be fair to remark that those who believe in the absolute commandments of God, to whom power belongs, may oppose democracy as the rule of the mortal and the fallible. As faith is not negotiable, its tenets cannot be changed through discussion and debate; so, if one believes that democracy is an abomination and against God's rule, one may not even ponder it.

Likewise, the logic of multiculturalism, as presented by Kymlicka, would not work for religion. Nothing about national or ethnic identity would make people from different races, ethnicities, or nationalities necessarily unable “to just get along,”^2^ except for past injustices that could be compensated for and wrongs that might be righted. By contrast, articles of faith may very well obstruct such “getting along” between adherents of different religions. And in question here are not the fine intricacies of religious doctrine that no average believer would really know or even care about, but the more basic knowledge received while growing up in a religious environment. For instance, who exactly was Jesus? Son of God, just a prophet, or a “false messiah”? As the answer will depend on one's faith and the matter cannot be resolved through reasoning or negotiation, it is best to leave the question to one's conscience without subjecting it to public debate and deliberation—a point to which we shall return.

While it is true that one is born into a religious community, one could convert, albeit with some difficulty, or one may simply not own up to the religious identity. By contrast, race (or gender) is not only immutable, biologically speaking, it is also a badge assigned to the wearer by others, sociologically speaking. Race is always relative, in terms of both the classification of physical attributes and the social meanings attached to them. Thus, even though the physical attributes may not be changed, racism lies in the classification and the attached social meanings, both of which may be changed. Religion is altogether different. It is not attributed from outside; one owns it. It does not consist of a set of imaginary social meanings; it is often inscribed into one's life-style, world-view, daily habits and rituals, ideas, and beliefs, which one either adopts or rejects. Unlike race or gender, the attribution of those social characteristics may be refuted by distancing and disavowal. Religion may indeed be an ascribed identity in premodern societies where communalism reign. But what about modern societies where people intermingle, marry, and procreate across religious lines? What communal identity do the children of mixed marriages have, other than those selected by themselves?

Modood complains of the “racialization” of religion by the majority culture in Europe through the attribution of unfair characteristics to *all* members of a community, such that Muslims are associated with terrorism, and so on (Modood, 2019, Ch. 3, 4, *passim*). It is true that only a very tiny fraction of all Muslims in Europe, and in the world, may be engaging in acts of violence in the name of Islam. Yet Modood himself seems unwittingly to contribute to this “racialization” by welcoming the establishment of corporate bodies that aim to represent Muslims collectively, such as the creation of the Muslim Council of Britain in the UK, the *Conceil Français du Culte Musulman* in France, and the *Deutsche Islam Konferenz* in Germany (Modood, 2013, Ch. 8; Modood, 2019, *passim*). Would it not make more sense to ideationally separate the collectivity from the unlawful behavior of occasional individuals? To illustrate the point, we might ask whether it would be a good idea to create a Gay Council of Britain, if religion is (as Modood suggests) on a par with sexual orientation as an ascriptive attribute. One may safely presume, however, that gays and lesbians would rather be left alone to live their lives without any interference by authorities. While instituting procedures to advise governments on proper policies would be worthwhile, fixing religion (or sexual orientation) as the primary identity of a group of citizens would weaken and impoverish their participation in civic life.

The responsibility of the democratic state is to preserve equality between citizens and hence to protect them from discrimination. The corresponding principle in the realm of religion is, simply, secularism. Yet, Modood demands special protection for religion by promoting arrangements that would not be contemplated for other marginalized communities. This may in the end defeat the purpose, with such bodies ending up as His/Her Majesty's Councils, alienating ordinary members of the community, regardless of their degree of identification with the religion so represented. This brings us to the second issue, that of policy.

### Multiculturalizing (moderate) secularism

Undoubtedly, individuals may derive their sense of dignity from their communal identities. The problem is when the state treats them not as independent citizens, but as representatives of certain well-defined and formally circumscribed communities. While it is necessary to acknowledge “cultural diversity” in order to avoid forcing everyone into a single mold defined by powerful elites, the solution should not be granting collective group rights, which only moves the assumption of homogeneity from the national to a subnational level. Granting rights to communities reinforces the collective identities and ignores the rights of persons as independent individuals. As Parekh (2000, p. 340) notes, a multicultural society is both “a community of citizens and a community of communities, and hence a community of communally embedded and attached individuals.” If these communities are inscribed into the political system, they become fixed and frozen. Defining individuals as members of such (imaginary) homogeneous communities and endowing those entities with rights, including a measure of legal autonomy, disempowers individuals against any possible oppressive practices within the communal hierarchy.

An example is offered by Mahajan (2017), who argues that Modood's concept of “moderate secularism” does not adequately address religious diversity. In India, she notes, there is a deeper recognition of communal identities. But after describing at length the arrangement by which India achieved what European secularism failed to achieve, that is, how India “chose to be inclusive and made a conscious effort to recognize and accommodate its religious diversity” (Mahajan, 2017, p. 81), she allows that the autonomy granted to minority communities in “personal [status] laws” has rendered the state unable to maintain gender justice (Mahajan, 2017, p. 82–83).

Or take the cases of Indonesia and Malaysia, which like India are seen as models of religious diversity and freedom, and where citizens are treated primarily as members of given communities rather than as autonomous individuals. In both nations citizens are obligated to identify with a religion that is officially recognized by the state and have it inscribed in their identity cards. The same practice also obtained in Greece and Turkey, where national identities are primarily based on religion, and was only recently lifted in both countries in the context of their dealings with the European Union and the European Court of Human Rights. Unlike these two countries, in Indonesia and Malaysia the institutional structure may appear to favor religious diversity and freedom, but “individual freedom of conscience, including conversion for instance, is thus circumscribed” (Sealy et al., 2022, p. 459).

Lebanon is another example where politics and citizenship rights are organized around confessional lines. Individual religious conversions are possible; but, for instance, a secular option is not available for concluding inter-sectarian civil marriages through a non-religious personal status law (Taşkin, 2021). Religious diversity is recognized and institutionally structured, but political rights and authority are vested in the leaderships of those officially recognized religious communities. In Lebanon, this political system has led to continuous tension and turmoil, including a prolonged civil war, with a persistent popular demand for a secular regime.

These examples show that a system built on a given number of religious communities, where a person's status is defined by their communal identity and political rights are only operative within the boundaries of the community, cannot enhance or encourage diversity that may grow within civil society. Such a system cannot be defined as liberal, because citizens are not seen as individuals with equal rights but only as members of specific religious groups. Considering that the concept of “multicultural” takes cultures as given, and of a certain number, the question also arises as to exactly how many cultures does “multi” imply? In other words, multiculturalism paradoxically restricts diversity.

The UK, despite an established church headed by the head of state, offers a significant counterexample. Notwithstanding its symbolic outward appearance as a “religious state,” one would be hard-pressed to define the UK as anything other than “secular” in terms of the state's treatment of its citizens. The UK had “blasphemy laws” against Christianity in its books, when the “Rushdie affair” broke out. The Muslim community demanded that the same prohibition also apply to blaspheming Islam, to which the state responded, albeit belatedly, by lifting blasphemy laws altogether (except in Northern Ireland). This was an exemplary normative respect for equal citizenship. But suppose the Muslim demand was met and then another religious community demanded the same kind of protection for their own sacred beliefs. Would there ever be an end to it or would the state have to draw the line somewhere; and, if so, where?

Still, if persons are not free-floating but always embedded in cultures, is there a solution to this dilemma of individual vs. community? There is, and it starts with the individual rather than the community within which they are presumed to be embedded. Naturally, how much dignity a person will derive from ascribed and how much from achieved characteristics will vary from person to person and from community to community. Besides, every person has multiple identities (or, rather, multiple aspects to their unique identity), and it is up to them to decide what community they wish to associate with. Instead of fixing and freezing “cultural” groups into the political system, diversity could be recognized through individual rights. Persons would then be free to form alliances and associations based on their changing social needs and/or identities. Any given person combines an indefinite number of socially significant characteristics: ethnicity, gender, race, language, religion, class position, professional status, age, physical ability, sexual orientation, political and philosophical orientation, and so on, in an open-ended list, because there may be dimensions of identity that are not socially significant or even conceivable now but may become so in unexpected ways in the future. Inscribing such answers into the political system will reduce the complexity of every person into a single dimension or a hierarchy of dimensions, which they have not necessarily chosen themselves. The answer to the question of policy should not start from the community defined top-down by the state, but from the citizens endowed with rights, who can create their own communities from the bottom-up.

Historically speaking, secularism was designed to address the issue of religious diversity in modern society (Akan, 2017). But there are those, such as Asad (1993, 2003), who think that secularism is the modern state's vehicle for sovereignty. If Tariq Modood's normative theory could be described as *semi-secularist*, Talal Asad's theory is completely *anti-secularist*, as we see next.

## Secularism as state sovereignty

It is useful to distinguish between theory and policy in Talal Asad's work as well, because while his theoretical arguments seem obscure and non-committal, his denunciation of the modern state's liberalism and secularism comes across clearly on political matters. Asad's two acclaimed books (Asad, 1993, 2003), rightly described by many as erudite, are also somewhat frustrating because they essentially engage in criticizing other writers' works, finding what he deems inconsistencies or other kinds of faults, mostly concerning minutia. Still, the postmodern thrust comes forth, strikingly resembling the modernization theory, only as its mirror reflection. Moreover, for all the hype about Foucault's genealogical method, Asad's arguments lack originality. This can be seen by comparing them with some examples from the literature.

### Religion and modernization

In *Genealogies of Religion*, Asad rejects Geertz's (1973) effort to propose a universal definition. Building on the postmodernist notion that structures come from discourse, he argues, “There cannot be a universal definition of religion, not only because its constituent elements and relationships are historically specific, but because the definition is itself the historical product of discursive processes” (Asad, 1993, p. 29). This is reminiscent of Smith's (1963) suggestion that the concept of “religion” as we know it today came into being in the context of the Enlightenment. Yet, Smith is not spared Asad's (2001, p. 221) criticism, because he had “nothing to say about ‘secularism.”' According to Asad, “‘religion' is a modern concept not because it is reified [as Smith suggests] but because it has been linked to its Siamese twin ‘secularism.”' This may appear as a trivial point, until one realizes that Asad's aim is to discard secularism, as we see below.

In the opening lines of the last chapter of *Formations of the Secular*, Asad (2003, p. 205) states: “At the beginning of this study I proposed that the modern idea of a secular society included a distinctive relation between state law and personal morality, such that religion became essentially a matter of (private) belief—a society presupposing a range of personal sensibilities and public discourses that emerged in Western Europe at different points in time together with the formation of the modern state.” This strikes one as nothing but a seemingly complex restatement of the classical theory of secularization.^3^ In this quote, Asad also refers to the role of the modern state in building secularism. Although buried in his obscure style, he argues that the modern state shaped religion by removing it from the public sphere and forcing it into the private, hence explaining the emergence of both religion and secularism simultaneously. But the role of the state in building secularism is not a new idea either. Keddie (1997), for example, has underlined the key role played by the state, in her objection to the conventional theory that attributes secularization to socio-economic processes. Balibar (2004, p. 355) points out, “there is no natural distinction between the political and the religious, but a historical one resulting from decisions that are themselves political.” Troper (2009, p. 2,561) makes a similar point about the sovereignty of the state, modern or otherwise: “sovereignty is the defining characteristic of the State.” He adds: “The State being sovereign, no subject matter escapes its power. Thus, influence over religion falls within the competence of the State, even the most secular one or even the State that apparently submits to religious law” (p. 2,574).

Unlike all these authors, however, Asad aims at a more general rejection of modernity, through what he conceives as a critique of liberalism and secularism. But his arguments confound modern, secular, and liberal in such a way that a critique of what is perhaps the inadequate liberalism of the modern state relative to its claims is offered as an argument for a denunciation of secularism. According to Asad, the liberal-secular state is at least as violent as the “fundamentalist” state that it supposedly opposes.

### Liberal violence

Asad's statements on this topic are more explicit but still frustrating because they often proceed by knocking down strawmen that he has set up. Secularism, he declares, is not necessarily a principle of peace and toleration in a diverse society, because “repeated explosions of intolerance … are entirely compatible (indeed intertwined) with secularism in a highly modern society” (Asad, 2003, p. 7). If, he argues, a secular state involves “a complex arrangement of legal reasoning, moral practice, and political authority,” it could not be described as “the simple outcome of the struggle of secular reason against the despotism of religious authority” (Asad, 2003, p. 255). It is not clear how one point follows from the other.

Asad's style almost takes the form of innuendo. For example: “Those who think that the motive for violent action lies in ‘religious ideology' claim that any concern for the consequent suffering requires that we support the censorship of religious discourse—or at least the prevention of religious discourse from entering the domain where public policy is formulated” (Asad, 2003, p. 11). What exactly is being said here? Obviously, religious ideology may or may not motivate violent action. But no one can claim that it never does. And, if it does so, it is incumbent upon the sovereign state (secular, liberal, or otherwise) to take action to prevent it. The state must also act when non-religious ideology does the same.

Yet Asad moves along, conflating liberalism, secularism, and modernity, from an almost anarchist-sounding perspective that aims to eschew the legal order: “What happens, the citizen asks, to the principles of equality and liberty in the modern secular imaginary when they are subjected to the necessities of the law?” (Asad, 2003, p. 6). But how can liberty and equality be maintained if there are no rules and regulations enforced by a state? At any rate, he concludes: “Liberalism is not merely the passion of civility … It claims the right to exercise power, through the threat and the use of violence, when it redeems the world and punishes the recalcitrant” (p. 60). One wonders what he might be proposing instead, until one realizes his longing for the premodern, pre-liberal order.

Creating further strawmen, Asad (2003, p. 100) tries to condemn secularism from another angle: “Only a secular legal constitution (so it is argued) can restrain, if not eliminate altogether, religious violence and intolerance toward religious minorities. This firm linking of institutional religion to cruelty has its roots in Western Europe's experience of religious wars and in the complex movement called the secular Enlightenment. But this perspective tends to overlook the devastatingly cruel powers of the twentieth century – Nazi Germany, Stalin's Russia, Imperial Japan, the Khmer Rouge, Mao's China—that were anything but religious, and the brutal conquests of African and Asian societies by European powers in the nineteenth century that had little to do with religion.” How does one account for and untangle this (perhaps intentional) muddle? No doubt, the modern state has been violent in cases of colonialism, as well as in these other instances, such as in Russia, China, and so on; but how exactly does that implicate secularism? Besides, it is *not* true that religion was not used as legitimizing ideology in modern European colonialism, nor is it the case that premodern religious states did not engage in violent imperialisms of their own.

There are numerous other examples of Asad's tendency to conflate freedom, equality, secularism, liberalism, and modernity, in response to which one could simply point out that not all modern states are liberal, even if they so claim, and not all freedoms are related to secularism. For instance, one cannot condemn secularism if there is inequality and lack of freedom in class or gender relations. Or, if a state must pursue law and order in order to protect democracy, one cannot infer that the state is exerting power through violence and then blame secularism for it. Or, one cannot condemn secularism by disputing the (allegedly secular) assumption that gives “rationality primacy in the constitution of the modern, secular subject” *via* the suggestion that persons are not necessarily rational but are subject to the influence of culture, emotions, and so on (Asad, 2003, p. 68–69).

Secularism as a normative political principle at least maintains the *promise* of freedom of thought and belief, even if it falls short in practice, whereas religious ideology (especially when sovereign) obviously does nothing of the sort. Asad would perhaps be justified if he were saying that the modern state violates its own assertion to be liberal when it engages in illiberal practices, as it occasionally (or frequently) does. But if he is holding the liberal (secular) state to its own standards and finding that it categorically fails, he may unwittingly be endorsing those standards despite his protests (cf. the similar point made by Laborde, 2017, p. 18). Manifest in Asad's critique of secularism is a general irony of postmodernist thought, which shares with modernization theory what Yack (1997) has called the “fetishism of modernities.” Like modernization theory, postmodernism overemphasizes the significance of modernity as a turning point in human history, only it does so in reverse. Whereas, for modernization theory modernity represents progress, freedom, equality, and other such good things, for postmodernism it represents absolutism, authoritarianism, stagnation, violence, and other such bad things. But the critique still takes place through the lenses introduced by modernity and in that sense implies its endorsement.^4^ Besides, it is not the only irony of postmodernist thought. The other has to do with its reaction to Eurocentrism.

### Postmodernism and Eurocentrism

The modern history of the West started with the transition to capitalism. But ideologically a myth of eternal superiority was created to explain what was historically contingent. A false continuity was thus proposed between such diverse episodes as ancient Athenian democracy, Christianity, and the Enlightenment along an unlikely unilinear history. The duality that Eurocentrism posits between the “civilized” Western (Christian) world and the “barbarian” Islamic world contradicts the universalistic ambitions of capitalism. Obviously not a product of Christianity, capitalism simultaneously unifies and divides the world. It aims and claims to homogenize the world, but instead generates uneven development. This contradiction is reflected in Eurocentric ideology, which is both universalist and relativist (Amin, 1989). Third World people are urged to emulate the West, while at the same time they are described as inherently incapable of it.

I have elsewhere argued that the origins of Islamism as a response to Western cultural imperialism lie in this contradiction. For Islamists, the Muslim world had been superior in premodern times. Their ideological reaction to Western culture thus stems from the emotional appeal of recapturing the imagined past glory, precisely at the time of the crisis of capitalist modernity, which found expression in the rise of postmodernism (Gülalp, 1992). But this reaction replicates the essentialism of Eurocentrism. In Said's (1978, p. 42) well-known account, “Orientalism” essentializes an “ineradicable distinction between Western superiority and Oriental inferiority.” Islamism shares the same assumption of an essential difference, only in reverse, positing Muslim superiority over the West (Al-Azm, 1981; Al-Azmeh, 1993).

Similar internal tensions and mirror-image reversals abound in Asad's analyses. On the question of contemporary Muslim presence in Europe, for example, Asad detects a confusion in European self-perception. He claims that the “problem of understanding Islam in Europe is primarily … a matter of understanding how ‘Europe' is conceptualized by Europeans” (Asad, 2003, p. 159), which can be seen in their reluctance to admit Turkey into the European Union: “In the contemporary European suspicion of Turkey, Christian history, enshrined in the tradition of international law, is being re-invoked in secular language as the foundation of an ancient identity” (p. 164). If this is true, then the problem must be originating not from secularism but from a lack of it, despite the European lip service. But Asad does not reach this conclusion. While contemplating whether historically Islam may be considered a part of European civilization, he first says, “There is a problem for any historian constructing a categorical boundary for ‘European civilization' because the populations designated by the label ‘Islam' are, in great measure, the cultural heirs of the Hellenic world—the very world in which ‘Europe' claims to have its roots” (p. 168). But only on the next page, he protests the notion of universalism on behalf of Muslims: The “Enlightenment's claim to universality” relies on the “idea that people's historical experience is inessential to them, that it can be shed at will” (p. 169). Moreover, he inexplicably twists this protest into a rejection of secularism. From the European point of view, he complains, “… Europe cannot contain non-Europe … immigrants in the grip of Islamic passions and ideas cannot live comfortably in the civilized institutions of *secular* Europe” (p. 171, *italics added*).

Regarding the source of secularism, Asad (2006a, p. 223) says on the one hand that “the historical connection of secularism with the formation of the modern nation-state is well-known.” But on the other he attributes “the construction of a secular space that begins to emerge in early modernity” to “the representation of the Christian God as being sited quite apart in ‘the supernatural' world…” (Asad, 2003, p. 27). Elsewhere, he replies affirmatively to a question about whether “a presumptively secular-universalist conception … [of] religion turns out to have its roots in a very specific Christian tradition” (Scott, 2006, p. 281). Here the reference is to Protestantism that considers belief and conscience to be a private choice, rather than communal tradition and practice. According to Asad (2006a, p. 247), this conception contrasts with Islam: “That conscience is a purely private matter at once enabling and justifying the self-government of human beings is a necessary (though not sufficient) precondition of modern secular ethics. The *shari'a*, in contrast, rejects the idea that the moral subject is completely sovereign… the individual's ability to judge what conduct is right and good (for oneself as well as for others) … [is] dependent not on an inaccessible conscience but on embodied relationships…” For Asad (1993, p. 42), there is a continuity between Christianity and secularism, which characterizes the West and contrasts with the Muslim world: “what appears to anthropologists today to be self-evident, namely that religion is essentially a matter of symbolic meanings linked to ideas of general order, … is in fact a view that has a specific Christian history.”

Like the Turkish Islamists who make postmodernist arguments, then, Asad is a postmodernist who makes Islamist arguments. According to him, Islam is a way of life, complete with communal traditions and practice, and cannot be privatized like Christianity. It must entirely encompass social and political life. Apparently, for Asad, secularism may be a problem in general, but it is especially a heavy burden on Muslims. In this emphasis on communal identity and collective difference, Asad joins the multiculturalists but goes further and aims to discard secularism altogether.

### Religion and politics

The concept of “Islamophobia,” originally introduced to draw attention to the systemic discrimination against Muslims in Europe, has mustered sympathy from progressive circles but also served globally to mobilize Islamist politics. Central in accounts of Islamophobia in Europe are the so-called “Rushdie affair,” which erupted in the UK in 1989, and the French Stasi Commission's recommendation to ban the use of Islamic headscarves in schools, which was then turned into law in 2004.

Asad (1993, p. 269–270), ever ready to conflate Western colonialism with liberalism and secularism, declares that “imperial power has made itself felt in and through many kinds of writing, not least the kind we call fiction,” and cites Salman Rushdie's *Satanic Verses* as an example. He also (indirectly) offers a psychological diagnosis on the author: “in translating a remembered childhood experience of repressive-parents-using-religious-rules into ‘religious repression', the adult subject has entered a discourse that already has high value in liberal secular culture” (p. 286, *fn*. 12). This is an odd remark not just because of the inappropriate insinuation, but because it makes one wonder how “religious repression” takes place if not through the acts of parents, relatives, vigilant neighbors, or the clergy or the state itself, bent on implementing (their own understandings of) religious rules.

Asad (1993, p. 291) also conflates the book with a weapon: “if the book's primary aim is to lampoon the sacred beliefs and practices of Muslim immigrants in Britain, then the literary devices employed in *The Satanic Verses* are entirely apt. Since these beliefs and practices are part of Muslim immigrants' contemporary social existence, their subversion requires a text that is a weapon.” The inference is that liberals ought not to have been so exercised with the death threat against Rushdie and the burning of his book.^5^ Their reaction, according to Asad (1993, p. 303), reveals “an aspect of the liberal concept of violence: for violence becomes a serious object of liberal concern only when something that they really value seems to be threatened.” Finally, finding a way to sneak secularism into the mix, he states in his closing words on the matter that “Muslims, Jews, Hindus, Buddhists, and Christians continue in our day to perpetrate acts of violence and cruelty. But it is the secular modern state's awesome potential for cruelty and destruction [as in the Holocaust] … that deserves our sustained attention …” (Asad, 1993, p. 306). This single-minded (but unjustified) assault on secularism indicates an ideological preoccupation rather than a scholarly interest.

Regarding the issue of headscarves, Asad (2006a, p. 218) claims to detect an internal contradiction of secularism, at least as practiced “in France [where] ‘religious' symbols are seen to collide with the state's representation of itself as essentially secular, but it is the state that decides which are religious symbols.” If the state is secular, he means, it should not be defining or dealing with religion or religious symbols or their manifestation by citizens (see also Asad, 2006b, p. 500). Several points may be made in relation to this charge.

First, the question of whether the French ban was justified does not correspond to a commendation or condemnation of liberalism and secularism in general.^6^ In other words, banning headscarf use does not meaningfully derive from normative secularism (Joppke, 2007; Bowen, 2010; Gülalp, 2013; Laborde, 2017).^7^ It has been noted, moreover, that the anxiety expressed in the Stasi report and the resulting legislation originated not from *laicité per se*, but from “the perception of an exceptional ‘threat' to French public order by Islamist groups” (Jansen, 2011, p. 10; see also Weil, 2004; Joppke, 2007; Bowen, 2010), a perception that cannot be dismissed as purely imaginary (Gülalp, 2019).

Second, Asad would have us believe that the state's practice of defining, determining and regulating religion from above is an indication of secularism. But consider Saudi Arabia or the Islamic Republic of Iran. Although these states have not removed religion from the political sphere to confine it to the private, they do what Asad accuses the modern-liberal state of doing. They define correct religion, enforce or limit religious practices, distinguish between proper and improper faiths, and discriminate against citizens of different faiths. Or, take Egypt, where the constitution cites Islamic Sharia as its source. Noting that headscarf use in schools was banned in both Egypt and (10 years later) France, Troper (2009, p. 2,562) observes, “in two very different countries … we find the State prohibiting the same type of religious behavior.”

The *premodern* Ottoman Empire likewise defined, shaped, and regulated religion by giving protected status to Jews and Christians as “people of the book,” while violently treating as “heretic” and “atheist” those non-Sunni Muslim groups such as the Shiite and the Alevi, along with the Zoroastrian and Manichaean. This policy derived from the priorities of state power, for all these “heretic” groups had Persian origins. After Arab lands were conquered by the Ottomans and the title of Caliphate was assumed, the entrenched Islamic identity of the empire generated a Sunni zealotry and led to the systematic suppression of the supporters of the Persian Safavid Empire (Barkey, 2008). As we see below, the Ottoman practice of recognizing or not recognizing a belief system as legitimate carried over into the republican period. This leaves us in a quandary. If the key concept that defines secularism is the state's power to determine the place of religion, then the Ottoman Empire must be as secular as the Kemalist Republic of Turkey. But if this is true, then the association of secularism with modernity and liberalism cannot be valid.

### Secularism or religious politics?

Ironically, the problems in Asad's theory are best revealed in the works of his followers. Exemplary are two books, Dressler's *Writing Religion* (Dressler, 2013) and Hurd's *Beyond Religious Freedom* (Hurd, 2015), which are presumably inspired by Asad's ideas but (perhaps inadvertently) serve to disprove them.^8^

Dressler brilliantly explains the transformation of what was known as the *Kizilbaş* community of the Ottoman Empire to the *Alevis* of present-day Turkey. Rightly attributing the twists and turns of this mutation to political priorities, he includes in the analytical narrative such diverse actors as the Ottoman state, Christian missionaries, Western anthropologists, Turkish nationalists, and secular Kemalists. He remarkably uncovers the “genealogical” thread; but the problem begins when he attempts to critique secularism by citing Asad's work. He says, for example, that secularist and Islamist discourses are alike, because both describe “the Bektashis and the Kizilbaş-Alevis as syncretistic,” serving to delegitimize them and to normalize the Sunni establishment. He goes on: “Modern Turkish secularism, or laicism (*laiklik*), is the best example. Its interest in controlling the content and boundaries of religion, and its authority to define what kinds of religious practices are legitimate in public spaces reflect the merger of Islamic modernism and political secularism” (Dressler, 2013, p. 219).

This is a puzzling argument, because in fact (*and* in Dressler's account) the mentioned policy began in the early Ottoman Empire and was eventually inherited by the Turkish Republic. With some ups and downs, assiduously detailed by Dressler, the policy of repudiating these groups or treating them as heretic sects has remained roughly constant all the way to the present. Dressler obviously knows but somehow fails to acknowledge this fact. To bolster his argument, he quotes (Dressler, 2013, p. 222) historian Kafadar's observation that clear boundaries could not be drawn between orthodoxy and heterodoxy in the times and regions of the empire that had not come under “such structures of authority as could define and enforce a ‘correct' set of beliefs and practices in the mode of learned Islam” (Kafadar, 1995, p. 73). This is true, but in the same paragraph Kafadar also points out that there was little need for “the Turco-Muslim polities of Western Asia … to be rigorously correct *until* the rise of the (Sunni) Ottoman—(Shi'i) Safavid rivalry” (*italics added*). Dressler concurs and states that it “was precisely the political confrontation between the Ottomans and the Safavids that helped—by denouncing the religious orientation of the respective other—to establish Sunni and Shiite standards of faith, respectively” (Dressler, 2013, p. 226).

Orthodox Sunni identity won decisive hegemony within the Ottoman Empire in the context of rivalry with the Safavid state of Iran. The conflict culminated in the battle of *Chaldiran* in 1514, resulting in Ottoman victory. Thereafter, non-Sunnis were perceived as collaborators of the neighboring rival Muslim empire and treated as more of an internal threat than non-Muslims. This policy was inherited by the Republic and non-Muslims were added into the mix, owing to the interreligious conflicts of the late nineteenth and early twentieth centuries. Considering the gap of four centuries between the battle of *Chaldiran* and the First World War, which marked the beginning of the end of the Empire and the rise of the Republic, one would think that Dressler's work serves to question rather than confirm Asad's theory.

Alevis are currently the largest religious minority in Turkey, with nearly 20 percent of the population, and have experienced continual persecution and discrimination throughout these centuries. Concealing their identity for safety among the Sunni majority, Alevis did not demand public recognition until very recently. Dressler does not address this question, as he is concerned with the designs of the power centers, but it is significant that the Alevi community began to assert a distinct religious identity only from the 1990s, in the context of a global politics of authentic identities (Göner, 2005).

Is the secular state responsible for the rise of religious identities? According to Hurd (2015), it is; and not in the sense of indirectly provoking a religious backlash, but directly through its mobilization of religious communities and political movements. With this unlikely idea, she complains of the rise of religious identities in politics, but cites the secular state as its source.

Hurd (2015, p. 7) describes her project as an attempt to dethrone religion “as a stable, coherent legal and political category” and rightly points out: “Religion does not stand outside or prior to other histories and institutions.” Her primary concern is the US foreign policy of “engagement with faith communities” to reach political ends through a language of “religious freedom.” She argues that this rhetoric serves neither religious nor other types of freedom, because defining individuals and groups in religious terms rules out other forms of affinities that may be equally or more relevant in people's lives. With such a categorization, “not only are particular hierarchies and orthodoxies reinforced, but dissenters, doubters, those who practice multiple traditions, and those on the margins of community are rendered illegible or invisible” (Hurd, 2015, p. 39).

These are important points, except that Hurd somehow relies on Asad's ideas to blame the secularism of the modern state for this mode of politics. But active engagement with faith communities is precisely what a secularist state must avoid. As she insightfully suggests, the policy of promoting religion and religious communities in foreign affairs may be a violation of the “establishment clause” of the US constitution's First Amendment (Hurd, 2015, p. 79*ff*). It follows that such misguided affairs as those in Afghanistan and elsewhere cannot be seen as an outcome of secularism. Hurd places the US International Religious Freedom Act of 1998 in this context and notes that this policy of promoting “religious freedom,” as she calls it, has developed especially after 9/11.^9^ But how can the secularism of the modern state be blamed for policies based on a piece of legislation that was passed two whole centuries after the formal institution of secularism?^10^ Clearly, the emergence of this policy at the precise historical juncture of the global weakening of the welfare state and the rise of identity politics in a context dominated by neoliberalism indicates the *decline* and not an eventual outcome of secularism.^11^ There is nothing wrong with religious freedom, and so no need to go “beyond” it, in Hurd's words, if it is granted to individuals as a human right. The problem is with the manipulation of religion or religious communities for political ends.

## Concluding thoughts: Learning from Thomas More

We have seen that both multiculturalists and postmodernists perceive secularism as a restraint on religion(s) and promote freedom *for* religion(s), rather than freedom *of* religion for the citizen. But while Modood's multiculturalist perspective proposes only a “moderate” secularism, Asad's postmodernist perspective implies a complete rejection of it, so that, one surmises, all social and political life may return to the premodern and pre-liberal warm embrace of religion. We have also examined the problems associated with each perspective. By way of conclusion, we now turn to an unexpected source for further insight into secularism.

Thomas More's famous book, *Utopia* (More, 1516), is known as an early depiction of a “communist” society, often inviting comparison with Marx's ideas, but his conception of “religious freedom” in that imaginary country is generally ignored. The modern concept of freedom of religion is usually attributed to John Locke, whose work on the matter (which appeared in 1689) came nearly two centuries after More's. It is surprising that More's ideas are rarely, if ever, invoked in debates on secularism and religious freedom.^12^ In fact, Thomas More's *Utopia* poses a challenge to those who conflate liberalism, individualism, modernity, and secularism. As a firm defender of Catholic orthodoxy, who was beheaded by Henry VIII in 1535 and canonized by the Pope four centuries later in 1935, More's utopian vision for freedom of religion long predates the Westphalian Treaty of 1648, the “liberal theory” of the eighteenth and nineteenth centuries, and the “modernization theory” of the twentieth.

In More's narrative, Utopus (the founder of Utopia), knew before his arrival to the island that religious quarrels sowed deep divisions that made it easy for him to conquer the island. After conquest, “he made a law that every man might be of what religion he pleased, and might endeavor to draw others to it by the force of argument and by amicable and modest ways … [and] that he ought to use no other force but that of persuasion. … This law was made by Utopus, not only for preserving the public peace, which he saw suffered much by daily contentions and irreconcilable heats, but because he thought the interest of religion itself required it” (More, 1516, p. 77–78).

More goes on: “Though there are many different forms of religion among them, yet all these, how various soever, agree in the main point, which is worshiping the Divine Essence; and, therefore, there is nothing to be seen or heard in their temples in which the several persuasions among them may not agree; for every sect performs those rites that are peculiar to it *in their private houses*, nor is there anything in the public worship that contradicts the particular ways of those different sects. There are no images for God in their temples, so that everyone may represent Him to his thoughts according to the way of his religion” (p. 83, *italics added*).

Thomas More's depiction strikingly resembles, though it predates by centuries, the modern notion of secularism and the vision of the “infidel” Ottoman sultan. In remarkable imagery, it reveals the essence of secularism, which is designed for the protection of freedom and diversity of religion. Religion is no doubt sacred for the believer, but only for the believer. So, what needs recognition and protection is not religion itself, but the right of the believer to hold it sacred, alone or in communion with others, freely but as a private matter.

The significance of the point about keeping religious faith private may be illustrated by citing a personal anecdote narrated by Tariq Modood, which indicates to him the significance of mutual “respect for religion,” but which may also be read as a demonstration of why faith must not be brought into public and political discourse. As a pupil at secondary school, Modood was urged by his father, a devout Muslim, to attend the daily Christian worship, from which he could be exempted if he so wished. His father noted that, at any rate, it was preferable to any other activity that was available. Modood (2019, p. 157–158) goes on to relate that, even though according to his father there was much overlap between the two faiths, “whenever it was said that Jesus was the Son of God, I should say to myself ‘No, he is not.”' The significance of the emphasis on “privacy” comes across in this anecdote. Clearly, he could not have expressed his own belief out loud. It would have caused unanticipated problems. By the same token, he should not have been subjected to this difficult experience of having to silently reaffirm his belief that differed from the majority. The whole ceremony should have been carried out in private places, as pointed out by Thomas More, and not in the public space of the school.

This is what secularists have in mind when they consider schools secular spaces. Ideas can be debated, but beliefs cannot. Faith is non-negotiable, if one is firm in their belief. It is, by definition, impervious to evidence and reason. It should have no place in (secular) schools, where reason must rule (which does not mean that individual pupils or students may not believe or manifest their belief as they wish), or in (secular) politics, where deliberation, negotiation, disputation, and compromise must rule (which, again, does not mean that ideas may not be inspired by faith). Faith, which could well be perfectly blind, cannot be more deserving of protection than thought, which could be brilliant, creative, reasonable, or simply banal or stupid. While one could easily debate and refute a thought, one would normally have difficulty to change one's own or someone else's faith. It is therefore incongruous to demand special protection for religion(s) while we constantly argue about differences in thought and opinion. What needs protection is not thought or faith itself, but the right to have them, without being subjected to any denigration or discrimination, so long as others are not somehow harmed by them.

## Author contributions

The author confirms being the sole contributor of this work and has approved it for publication.

## Footnotes

^1^Briefly defined, secularism is a political principle that aims to guarantee citizens the right to freedom of conscience and religion, as spelled out in international human rights documents, and entails the institution of a political space separate from and independent of religions for the purpose of negotiating common issues and areas of concern, so that the social and political needs of all religious and irreligious members of society may be met.

^2^The memorable phrase of Rodney King, the African-American motorist who was subjected to police brutality in Los Angeles, back in 1992.

^3^Casanova (1994, p. 15), who is likewise criticized by Asad, offers the following pithy definition: “If before, it was the religious realm which appeared to be the all-encompassing reality within which the secular realm found its proper place, now the secular sphere will be the all-encompassing reality, to which the religious sphere will have to adapt.” See also the definition previously offered by economic historian Tawney (1936, p. 279), in his classic materialist revision of Weber's theory on the Protestant foundations of capitalism: “Religion has been converted from the keystone which holds together the social edifice into one department within it…” resulting in “a dualism which regards the secular and the religious aspects of life, not as successive stages within a larger unity, but as parallel and independent provinces, governed by different laws, judged by different standards, and amenable to different authorities.” These authors speak of structures rather than discourse.

^4^This lack of self-awareness contrasts with Marx's critique, who as a modernist took modernity's promises of freedom and equality seriously but believed that they could not be fulfilled under conditions of capitalism and so further movement along modernization was necessary to transcend those conditions.

^5^Postmodernism has been described as the “retreat of intellectuals” from an earlier revolutionary position against capitalism to the more comfortable and fruitful arena of language and culture wars (Petras, 1991). Matching a book with a weapon must be the highest fantasy of postmodernist intellectuals who find discourse at the source of structural power relations (One is disturbed to ponder all this, considering the recent assault on Salman Rushdie that seriously injured him). Compare Asad's perspective with the infinitely more thoughtful and sober discussion of the Rushdie affair by Parekh (2000, Ch. 10), who nonetheless cannot escape Asad's (1993, p. 280) customary quibbling and gets accused (unfairly in my judgment) of disparaging Muslims by speaking of their “empty lives” as immigrants.

^6^For some unclear reason, Asad (2006b, p. 518) keeps repeating: “I should stress again that my concern here is not with defending the right to veil.” As a (liberal) secularist, however, I for one would readily admit that right. I also wonder what Asad would say about the rights of women in Iran who are struggling to remove the veil forced on them by the Islamic regime. Incidentally, it is saddening to see that the uprising in Iran does not receive much attention from Western democrats and leftists, who possibly fear accusations of Islamophobia or, worse, of inciting Western intervention.

^7^The French parliament's middle-of-the-road solution to ban all “ostentatious” religious signs, apparently in order to avoid an anti-Muslim appearance, violates the freedom to “manifest” religion or belief, enshrined in the European Convention on Human Rights (Article 9), and detailed by the European Court of Human Rights in the ground-breaking case of *Kokkinakis v. Greece* (ECtHR, 14307/88, 25 May 1993).

^8^A different kind of example is Mahmood's *Religious Difference in a Secular Age* (Mahmood, 2016), which I cannot discuss here for lack of space. Unlike the others, Mahmood is Asad's closest follower, and in her book Asad's theses are no longer concealed behind complex and obscure expressions, or repeated disclaimers, but laid bare and taken to their (disturbing) logical conclusions, such that all problems (or those so defined by her) are attributed to secularism, whether the connection can be established or not. Critical commentaries include Makdisi (2016) and Abu-Odeh (2017).

^9^Hurd (2015, p. 140, n. 7) points out: “The National Association of Evangelicals, which led the campaign for IRFA's passage in the 1990s, describes religious freedom as a God-given human right that occupies a privileged position above other rights claims”.

^10^It must be noted that the idea comes from Asad (2003, p. 146) himself, who characterizes the 1998 Act in these terms.

^11^On the Alevi question in Turkey, Hurd asserts that, rather than serving religious freedom, the European Court of Human Rights “construes Alevism as a non-Sunni Muslim minority sect in need of legal protection” (Hurd, 2015, p. 96), as though it were not the Alevis themselves who have applied to the European Court with grievances. This directly conflicts with Saba Mahmood's notion that secularism necessarily entails religious majoritarianism and ignores the needs of minorities. It is amazing how the ambiguity of Asad's thesis allows it to stretch in any direction that one pulls. You are damned if you give rights to religious minorities and damned if you don't.

^12^Kessler's (2002) work is an exception in this regard, but has likewise gone unnoticed.
